# Kinesin-2 and kinesin-9 have atypical functions during ciliogenesis in the male gametophyte of *Marsilea vestita*

**DOI:** 10.1186/s12860-016-0107-7

**Published:** 2016-07-16

**Authors:** Erika J. Tomei, Stephen M. Wolniak

**Affiliations:** Department of Cell Biology and Molecular Genetics, University of Maryland at College Park, College Park, MD 20742 USA

**Keywords:** Kinesin-2, Kinesin-9, Ciliogenesis, Cytokinesis, Intraflagellar transport, Basal bodies

## Abstract

**Background:**

Spermatogenesis in the semi-aquatic fern, *Marsilea vestita*, is a rapid, synchronous process that is initiated when dry microspores are placed in water. Development is post-transcriptionally driven and can be divided into two phases. The first phase consists of nine mitotic division cycles that produce 7 sterile cells and 32 spermatids. During the second phase, each spermatid differentiates into a corkscrew-shaped motile spermatozoid with ~140 cilia.

**Results:**

Analysis of the transcriptome from the male gametophyte of *Marsilea* revealed that one kinesin-2 (MvKinesin-2) and two kinesin-9 s (MvKinesin-9A and MvKinesin-9B) are present during spermatid differentiation and ciliogenesis. RNAi knockdowns show that MvKinesin-2 is required for mitosis and cytokinesis in spermatogenous cells. Without MvKinesin-2, most spermatozoids contain two or more coiled microtubule ribbons with attached cilia and very large cell bodies. MvKinesin-9A is required for the correct placement of basal bodies along the organelle coil. Knockdowns of MvKinesin-9A have basal bodies and cilia that are irregularly positioned. Spermatozoid swimming behavior in MvKinesin-2 and -9A knockdowns is altered because of defects in axonemal placement or ciliogenesis. MvKinesin-2 knockdowns only quiver in place while MvKinesin-9A knockdowns swim erratically compared to controls. In contrast, spermatozoids produced after the silencing of MvKinesin-9B exhibit normal morphology and swimming behavior, though development is slower than normal for these gametes.

**Conclusions:**

Our results show that MvKinesin-2 and MvKinesin-9A are required for ciliogenesis and motility in the *Marsilea* male gametophyte; however, these kinesins display atypical roles during these processes. MvKinesin-2 is required for cytokinesis, a role not typically associated with this protein, as well as for ciliogenesis during rapid development and MvKinesin-9A is needed for the correct orientation of basal bodies. Our results are the first to investigate the kinesin-linked mechanisms that regulate ciliogenesis in a land plant.

**Electronic supplementary material:**

The online version of this article (doi:10.1186/s12860-016-0107-7) contains supplementary material, which is available to authorized users.

## Background

Spermatogenesis in the semi-aquatic water fern, *Marsilea vestita*, is a rapid, synchronous process that produces motile gametes in only 11 h [1]. Similar to other rapidly developing systems, spermatogenesis in male gametophytes of *Marsilea* is controlled at a post-transcriptional level. Virtually all the RNA required for development is present in the dry spore at the time of its rehydration and almost no additional transcription is needed for spermiogenesis to reach completion [2–4]. Rapid development is dependent on the unmasking of pre-mRNAs [5], which are stored in dry spores within nuclear speckles [6], and then, the processing of these pre-mRNAs for translation [7]. Spermatogenesis begins when microspores are exposed to water. Shortly after hydration, the gametophyte initiates a developmental program that culminates with the production of 32 corkscrew-shaped spermatozoids, each with ~140 cilia [8–11]. Development can be divided into two phases. The first phase consists of a series of nine mitotic division cycles that produce 32 spermatids that are surrounded by seven sterile cells. All divisions are complete approximately 5 h after microspore hydration. During the second phase, only the spermatids undergo drastic morphological changes and differentiate into motile, corkscrew-shaped spermatozoids [4, 12].

This unusual shaping of the gamete is achieved through the elongation and coiling of the nucleus and mitochondria along a coiled ribbon of crosslinked microtubules. Basal bodies, already formed *de novo*, are placed in two rows at regular intervals along the dorsal face of the microtubule ribbon to become the sites of ciliogenesis [8, 11]. At first, basal bodies are oriented so cilia diverge away from each other and are parallel to the plasma membrane of each spermatid. Near the end of spermiogenesis, the basal bodies rotate 90° so that the ciliary axonemes protrude vertically from the microtubule ribbon and nuclear coil [13]. At 9.5 h of development, an extension of cytoplasm begins to grow around the anterior end of each spermatid and eventually fuses together to surround each cell. This creates an internal, but extracellular space that contains the microtubule ribbon and organelle coil plus all of the cilia. Upon release from the microspore, each spermatozoid breaks free from the surrounding cytoplasmic extension and leaves behind a thin vesicle-like structure [11]. The ciliary axonemes have the typical 9 + 2 architecture [13] found in motile organisms and spermatozoids are able to swim towards the megaspore for fertilization.

We are interested in the processes that regulate spermatid differentiation and ciliogenesis during male gametophyte development in *Marsilea*. Proteins important for ciliary assembly and function are moved to the distal ends of forming axonemes by intraflagellar transport (IFT) involving members of the kinesin-2 family. Heterotrimeric kinesin-2 consists of a kinesin-2α, a kinesin-2β, and a kinesin associated protein (KAP) that regulates cargo binding [14]. In *Chlamydomonas*, heterotrimeric kinesin-2 is necessary for IFT [15–18]. Kinesin-2 can also function as a homodimer of two kinesin-2γ subunits. Kinesin-2γ, also known as OSM-3 or KIF17, has a distinct role in assembling sensory cilia that is separate from heterotrimeric kinesin-2 [19–24]. Although the most common mechanism for axonemal assembly is dependent on IFT function, *Plasmodium falciparum* and the sperm flagella of *Drosophila* are able to build motile cilia and flagella using IFT-independent mechanisms. In this case, cilia are assembled in the cytoplasm [25–27]. Ciliogenesis in *Marsilea* does not occur in this way; instead, cilia are assembled in growing membrane extensions from basal bodies that are positioned along a microtubule and organelle coil. Therefore, we anticipated that ciliogenesis in *Marsilea* is reliant on IFT-dependent mechanisms of transport and assembly. In support of this idea, we found a variety of transcripts encoding IFT-associated proteins (kinesin-2, dynein-1b, IFT-A, and IFT-B subcomplex proteins) in the assembled transcriptome from the male gametophyte of this organism [7].

Phylogenetic analysis of kinesin-9 shows the existence of two subfamilies, kinesin-9A and kinesin-9B. Kinesin-9A includes KIF9 and kinesin-like protein 1 (KLP1), while kinesin-9B includes the KIF6 protein [28, 29]. Kinesin-9A is localized to the central pair of microtubules in the *Chlamydomonas* axoneme [30] and is necessary for motility in both *Chlamydomonas* [31] and *Trypanosoma brucei* [29]. Kinesin-9A appears to be important for motility by regulating the activity of flagellar dynein [31] and by interacting with Hydin, which is necessary for motility in algae [32], trypanosomes [33], and mice [34]. Less is known about the function of kinesin-9B; however, in *T. brucei*, KIF9B localizes to basal bodies and the flagellum where it is necessary for the construction of the paraflagellar rod [29].

Although the processes that regulate the structure and function of cilia are highly conserved, not all organisms make ciliated cells. This is most pronounced during the evolution and adaption of land plants from green algae. Conifers and angiosperms never make any ciliated cells, while lower plants known as embryophytes, including the ferns, mosses, liverworts, and certain members of the gymnosperms (*e.g*., *Ginkgo biloba* and the cycads) only produce cilia in their male gametes. In conjunction with the loss of cilia in the ‘higher’ organisms, the reduction [35], or complete absence, of proteins important for ciliogenesis and motility is also observed. For example, comparative analyses of the kinesin family has shown that kinesin-2, kinesin-9, and the more recently identified, kinesin-‘orphan’ III (also referred to as kinesin-16) and kinesin-17, are only found in organisms that are ciliated at some point during the life cycle [28, 36]. Members of these kinesin families can be found in ciliated plants such as *Chlamydomonas*, the moss *Physcomitrella*, and the water fern *Marsilea*, except kinesin-17, which is only present in *Chlamydomona*s [28, 36–38]. The genome of *Arabidopsis*, a flowering plant that is never makes ciliated cells, contains none of these kinesins [39]. Due to the reduction of cilia and IFT proteins in land plants, the majority of research on ciliogenesis in plants has been conducted in *Chlamydomonas*. It is somewhat ironic that the conserved 9 + 2 microtubule organization of motile axonemes was first observed in the spermatozoid of a fern [40].

Here, we are using spermatogenesis in *Marsilea* as a model for ciliogenesis. This provides us the unique ability to study the construction, organization and motility of ciliary apparatus produced in gametes of a land plant. Moreover, this gametohyte provides insights on the mechanisms that evolved to regulate *de novo* ciliogenesis in specialized cells of otherwise nonmotile organisms. Transcripts that encode members of the kinesin-2 and kinesin-9 families were selected as targets for our studies on the regulation of ciliogenesis in spermatids of *Marsilea*. mRNAs that encode kinesin-2 and kinesin-9 increase in abundance during the stage of development associated with spermatid shaping and ciliogenesis [38]. These late rises in transcript abundance led us to suspect that kinesin-2 and kinesin-9 may play critical roles for spermatid differentiation and ciliogenesis. Unlike many other systems, the male gametophyte of *Marsilea* has only one kinesin-2 that is apparent in its transcriptome. It is most similar to the kinesin-2 found in *Physcomitrella* and is divergent from the typical heterotrimeric kinesin-2 associated with IFT. In this study, we show that MvKinesin-2 is required for two separate events in this gametophyte. It is necessary for cytokinesis in spermatogenous cells and is also important for regulating the length of cilia during later phases of spermatid maturation. The *Marsilea* gametophyte has two transcripts that encode members of the kinesin-9 family; one is similar to the kinesin-9A and the other is most like kinesin-9B. In the gametophyte, we show that MvKinesin-9A is involved in the proper positioning of basal bodies that are required for ciliogenesis and it is necessary for motility. MvKinesin-9B is needed for the timely differentiation of motile spermatozoids in the rapidly developing gametophyte.

## Results

### Characterization of the kinesin-2 and kinesin-9 family in *Marsilea*

After conducting RNAseq with poly(A+)-RNA isolates obtained from male gametophytes of *Marsilea* during different intervals of development, we were able to assemble a transcriptome *de novo* [41], and calculate changes in transcript abundance that occur during different phases of spermatogenesis. We searched our combined reference transcriptome for members of the kinesin-2 and the kinesin-9 families using *Physcomitrella* and *Chlamydomonas* kinesins-2 and kinesin-9 sequences (Additional file 1). We used these kinesins as the base of our search because the entire kinesin family in *Physcomitrella* has recently been identified [37] and because *Physcomitrella* and *Chlamydomonas* are two of the more closely related ciliated species to *Marsilea* with an annotated kinesin family. We then verified our results by comparing them to the 56 kinesin-like transcripts that were previously identified in our transcriptome [38]. Our search identified one candidate kinesin-2 (MvKinesin-2) transcript and two candidate kinesin-9 (MvKinesin-9A and MvKinesin-9B) transcripts (Additional file 1).

**Fig. 1:**
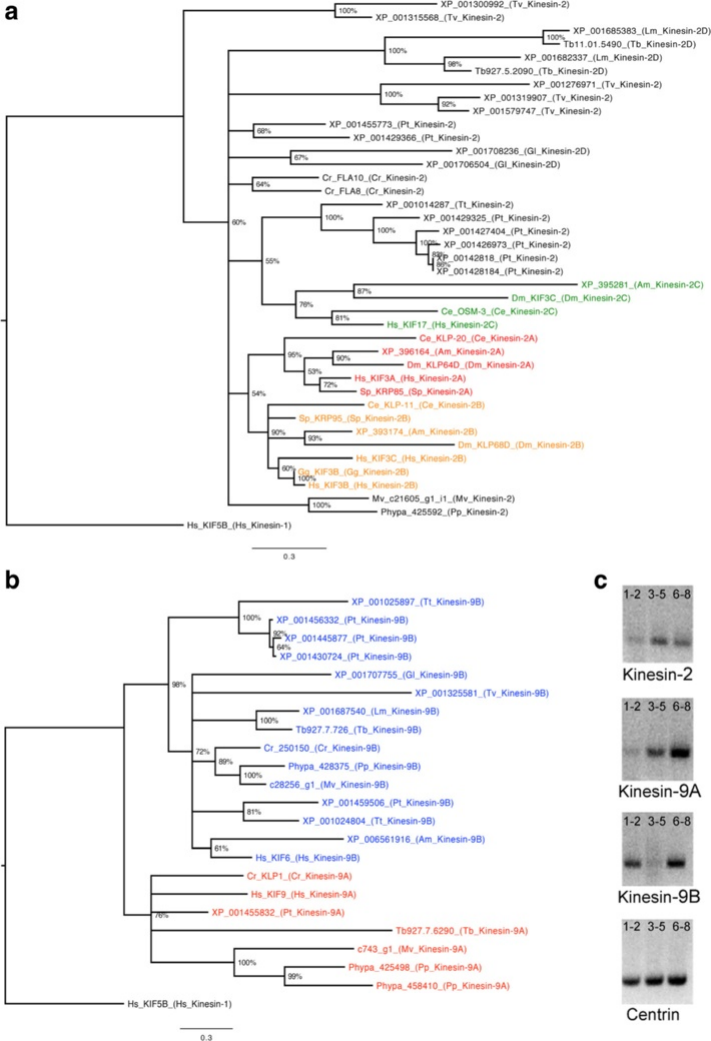
Characterization of the kinesin-2 and kinesin-9 families in *Marsilea*. **a**–**b** Maximum likelihood trees were constructed using the motor domain from well-established kinesin-2 and -9 sequences. The motor domain from kinesin-1 (Kif5) was used as an out-group for these analyses. **a** Kinesin-2 motors can be separated into three subfamilies; kinesin-2α (*red*), -2β (*orange*), and -2γ (*green*). There are also many kinesin-2 sequences that do not correspond to any well-supported subfamily. The kinesin-2 motor in *Marsilea* (MvKinesin-2) is most similar to the kinesin-2 in *Physcomitrella* and does not fall into a kinesin-2 subfamily. **b** Kinesin-9 can be separated into two well-supported subfamilies, kinesin-9A (*red*) and kinesin-9B (*blue*). *Marsilea* has one kinesin-9A (MvKinesin-9A) and one kinesin-9B (MvKinesin-9B). **c** An increase in MvKinesin-2, -9A, and -9B mRNA abundance was detected using RT-PCR against poly(A+)-RNA isolated at 1–2, 3–5, and 6–8 h of gametophyte development. The abundance of centrin mRNA does not change during development and was used as a control

Unlike other species, there is only one kinesin-2 present in *Physcomitrella* [37] and *Marsilea*. The translated kinesin-2 sequence from *Marsilea* (MvKinesin-2) shows that MvKinesin-2 recognizes members of the kinesin-2 family from other ciliated eukaryotes. Reciprocal Blastp analysis shows that MvKinesin-2 recognizes CrFLA8 (XP_001697037) and CrFLA10 (XP_001701510) with an e-value of 0.0. Phylogenetic analysis of the motor domain of established kinesin-2 s in plants and animals (Additional file 2) demonstrates that the single kinesin-2 motor in *Marsilea* cannot be placed in the standard kinesin-2α (-2A, shown in red), -2β (-2B, shown in orange), or -2γ (-2C, shown in green) subgroups (Fig. 1a). This is similar to our findings for both FLA10 and FLA8 from *Chlamydomonas* and for kinesin-2 from *Physcomitrella*. MvKinesin-2 is most similar to the kinesin-2 in *Physcomitrella* (Fig. 1a). This is not surprising since the only ciliated cells in *Marsilea* and *Physcomitrella* are their male gametes. It is unclear whether this kinesin functions as a homodimer, a heterotrimer with yet unidentified partners, or as a unique single protein.

The male gametophyte of *Marsilea* has two transcripts that encode kinesin-9-like proteins. Phylogenetic analysis of the motor domain of established kinesin-9 s in plants and animals (Fig. 1b, Additional file 2) demonstrates that the kinesin-9 s in *Marsilea* can be separated into the kinesin-9A (red) and kinesin-9B (blue) subfamilies (Fig. 1b). This provides further evidence for the existence of two distinct kinesin-9 subfamilies. Reciprocal Blastp analysis shows that translated kinesin-9A (MvKinesin9A) and kinesin-9B (MvKinesin9B) sequences in *Marsilea* recognize other members of the kinesin-9 family. MvKinesin9A is similar to CrKLP1 (XP_001701617) and TbKIF9A (XP_846252) with e-values of 4e-112 and 8e-50, respectively. MvKinesin9B recognizes CrKIF9B (Cre01.g036800.t1.1) and TbKIF9B (XP_846346) with e-values of 0.0 and 6e-89, respectively.

MvKinesin-2, MvKinesin9A, and MvKinesin9B have typical kinesin motor and ATP-binding domain architectures including the P-loop, switch I, and switch II motifs (Additional file 3). Outside of this highly conserved motor domain, MvKineisn-2, MvKinesin9A, and MvKinesin9B show a similarity to counterpart kinesins in *Physcomitrella* and *Chlamydomonas*, however, large insertions and gaps exist in the non-motor regions for each of these alignments (Additional file 3).

RT-PCR using poly(A+)-RNA isolated at 1–2 h, 3–5 h, and 6–8 h of development shows that MvKinesin-2, MvKinesin-9A, and MvKinesin-9B transcripts increase in abundance from the 1–2 h to the 6–8 h time intervals of gametophyte development (Fig. 1c). This pattern of transcript availability and abundance suggests that kinesin-2 and kinesin-9 transcripts are unmasked, spliced and polyadenylated prior to the time interval associated with ciliogenesis during gametophyte development [4]. It is unclear why kinesin-9B transcripts decrease during 3–5 h time interval. We have seen in our transcriptome analysis that the middle time point of development (3–5 h post hydration) appears to be a transition time between the early stage of development, marked by cell division, and the later portion of development, which is dedicated to spermatid differentiation and ciliogenesis. GO terms that are enriched during this transition time includea variety of nucleases, and proteasome and ubiquitin ligase components. It is possible that kinesin-9B transcripts and proteins are destroyed during this time period of development, and then additional kinesin-9B transcripts are unmasked, processed and translated for subsequent kinesin-9B functions during ciliogenesis.

### Kinesin-2 and kinesin-9 are involved in different aspects of spermatogenesis

**Fig. 2:**
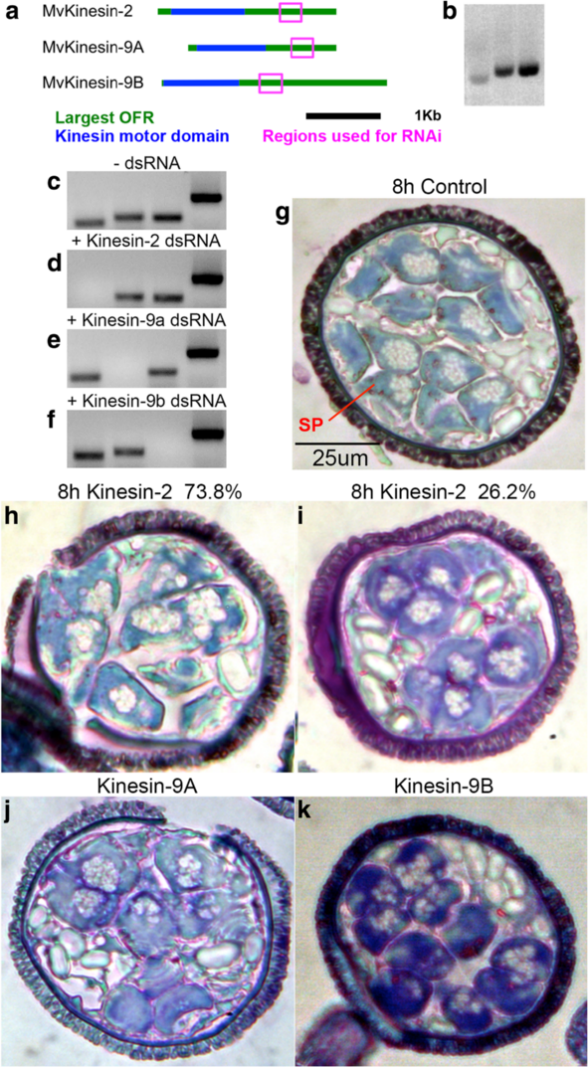
Kinesin-2 and kinesin-9 are involved in spermatogenesis. **a** Unique 350–400 nt regions used for constructing dsRNA. **b** dsRNA constructed from MvKinesin-2, -9A, and -9B, respectively, using poly(A+)-RNA isolated at 8 h as a template. **c** RT-PCR for MvKinesin-2, MvKinesin-9A, MvKinesin-9B, and centrin respectively, in the absence (-) of any dsRNA at 8 h of development. **d**–**f** RT-PCR for MvKinesin-2, MvKinesin-9A, MvKinesin-9B, and centrin, respectively, in the presence (+) of (**d**) MvKinesin-2 dsRNA, (**e**) MvKinesin-9A dsRNA, or (**f**) MvKinesin-9B dsRNA. Each transcript cannot be detected after its knockdown. Centrin mRNA does not change after the addition of various kinesin dsRNAs. **g** Untreated, control microspores developed for 8 h, embedded in methacrylate, sectioned, and stained with TBO. Spermatogenous (SP) and jacket (**j**) cells can easily be distinguished from each other. Sectioned microspores treated with (**h**–**i**) MvKinesin-2, (**j**) MvKinesin-9A, and (**k**) MvKinesin-9B dsRNA and stained with TBO

To determine the function of these kinesins during spermatogenesis, we performed RNAi knockdown experiments, treating gametophytes at the time of spore hydration with dsRNA. To do this we transcribed and constructed dsRNA from unique regions of each kinesin (Fig. 2a–b). It was important to use unique regions to make dsRNA in order to prevent broad silencing of off-target sequences and to ensure that only one kinesin was being silenced. The effectiveness of RNAi was measured using RT-PCR for each transcript after knockdown at 8 h of development. In each case, the presence of the transcript could not be detected after its individual knockdown, while the presence of other kinesin and centrin mRNAs remained unchanged (Fig. 2c–f). Microspores were grown for 8 h, fixed, embedded in methacrylate, and sectioned. The sections were then stained with toluidine blue (TBO) and examined with brightfield microscopy to observe broad morphological changes in development that might have resulted from the knockdown.

After 8 h of development in untreated gametophytes, all cell divisions are complete and spermatids can be clearly distinguished from jacket cells by their size, location within the microspore, shape, and staining pattern. Jacket cells are located towards the outside of the microspore near the microspore wall and contain starch filled plastids. Jacket cells do not contain very much mRNA or proteins and therefore do not stain intensely with TBO. During this stage of development, each spermatid begins to differentiate into a motile spermatid by forming a coiled nucleus and microtubule ribbon, creating a slightly boomerang shaped cell (Fig. 2g) [11].

Spermatogenous cells can clearly be distinguished from jacket cells in MvKinesin-2 knockdowns; however, in the majority of gametophytes (79/107; 74 %) spermatogenous cells are larger than normal and do not have a consistent size and shape compared to controls. This suggests that one or more cell division cycles have been skipped or failed to reach completion (Fig. 2h) [42]. In a minority (28/107; 26 %) of these knockdowns, spermatogenous cells are the correct size and shape and appear to be morphologically normal (Fig. 2i).

In MvKinesin-9A knockdowns, spermatogenous cells were the same size and shape as controls, suggesting normal progression through all of the cell division cycles (Fig. 2j). However, a distinct phenocopy was observed with knockdowns of kinesin-9B. In these gametophytes, spermatogenous cells appear larger than controls and the cells are rounded. This suggests that nuclear elongation failed to occur. Thus, while cell divisions have proceeded normally, differentiation is aberrant (Fig. 2k).

### Spermatid differentiation is incomplete after knockdown of MvKinesin-2, MvKinesin-9A, and MvKinesin-9B

**Fig. 3:**
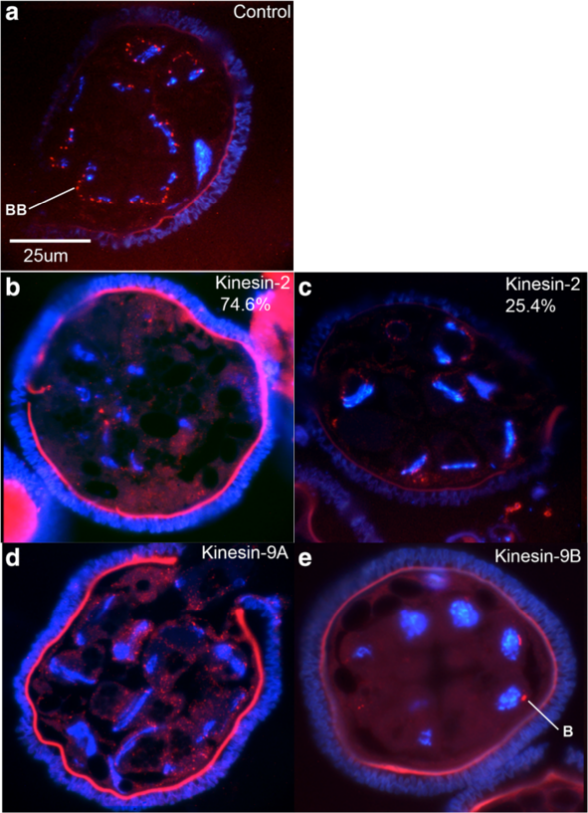
Spermatid differentiation is incomplete after silencing. **a** Untreated sectioned microspores developed for 8 h and labeled with anti-centrin Ab (*red*) and DAPI (*blue*). Centrin stains basal bodies (BB) and DAPI labels the nuclear coil. Sectioned 8 h microspores treated with (**b**–**c**) MvKinesin-2, (**d**) MvKinesin-9A, and (**e**) MvKinesin-9B dsRNA. **b** Centrin staining is diffuse and there are very few centrin aggregates that resemble basal bodies. The nuclear coil is not properly formed. **c** A small percentage of MvKinesin-2 knockdowns have a normal pattern of anti-centrin and nuclear staining. **d** Centrin staining is found throughout each spermatid and aggregates that resemble basal bodies are not localized at regular intervals adjacent to the nuclear coil. Most cells have a nuclear coil that appears similar to controls. **e** The blepharoplast (**b**), a centrosome like particle, is visible. Nuclei in these spermatids have failed to coil and differentiation has not occurred

In order to assess altered patterns of spermatid maturation, we fixed and sectioned gametophytes that had undergone silencing of MvKinesin-2, MvKinesin-9A, or MvKinesin-9B. We stained the methacrylate sections of fixed gametophytes with DAPI to observe nuclear elongation and labeled them with anti-centrin antibodies to observe the presence and distribution of basal bodies in the spermatids [5]. During normal spermatid differentiation, the basal bodies become situated at regular intervals along the microtubule ribbon and the nuclear coil where they serve as templates for the growth of ciliary axonemes (Fig. 3a). By observing the distribution centrin protein in kinesin knockdowns, we are able to make conclusions about the formation and localization of basal bodies in these cells [3, 5, 7].

In each knockdown, the appearance and placement of basal bodies is altered. At 8 h of development, anti-centrin staining in the majority (85/114; 75 %) of MvKinesin-2 knockdowns is diffuse and there are few, if any, aggregates of centrin that resemble typical basal body staining in spermatogenous cells. Some larger centrin aggregates that possibly resemble the blepharoplast can be found in spermatogenous cells (Fig. 3b). DAPI staining in these cells is also distinctly different from controls and it is apparent that the nuclear coil is not properly formed at this stage (Fig. 3b). Just as a minority of microspores treated with dsRNA complementary to MvKinesin-2 appeared to proceed through development normally when observed with TBO, a similar percentage (29/114; 25 %) also have normal centrin and DAPI staining (Fig. 3c). These microspores were either not affected by RNAi or it is possible that there are two separate phenocopies of MvKinesin-2 knockdown during spermatogenesis in *Marsilea*.

In MvKinesin-9A knockdowns, we observed aggregates of centrin staining that resemble basal bodies. The presumptive basal bodies become localized in areas of the spermatids where they normally are not seen. Later in development, these basal bodies become positioned at irregular intervals around the cell periphery; they are not restricted to the anterior (coiled) portion of the spermatid, as they would be in normal cells. DAPI staining in these cells shows a normally elongated nucleus (Fig. 3d). Therefore, the elongation stage of spermatid differentiation is occurring, but there are anomalies in the localization of basal bodies in these cells.

MvKinesin-9B knockdowns show large aggregates of centrin protein (Fig. 3e) that resemble blepharoplast particles. The blepharoplast is a cytoplasmic particle that forms during the last spermatogenous cell division. During the last mitotic division, the blepharoplast functions like a centrosome at the spindle pole, though it lacks any organized centrioles. As the spermatids are being formed, the blepharoplast disappears and then reforms to serve as a site for *de novo* basal body assembly [1]. In this knockdown, no further maturation of the blepharoplast (for basal body formation) was observed. Spermatogenous cells in MvKinesin-9B knockdowns exhibit nuclei that are round in shape (Fig. 3e), while normal spermatids undergo nuclear elongation and coiling [11]. This pattern of centrin immunolabeling and DAPI nuclear staining is characteristic of arrest at a stage of development preceding basal body formation in spermatids, and suggests that either spermatid maturation has stopped early in gamete differentiation or that the differentiation process has been slowed substantially.

### Silencing of MvKinesin-2, MvKinesin-9A, and MvKinesin-9B cause defects in ciliogenesis and motility

**Fig. 4:**
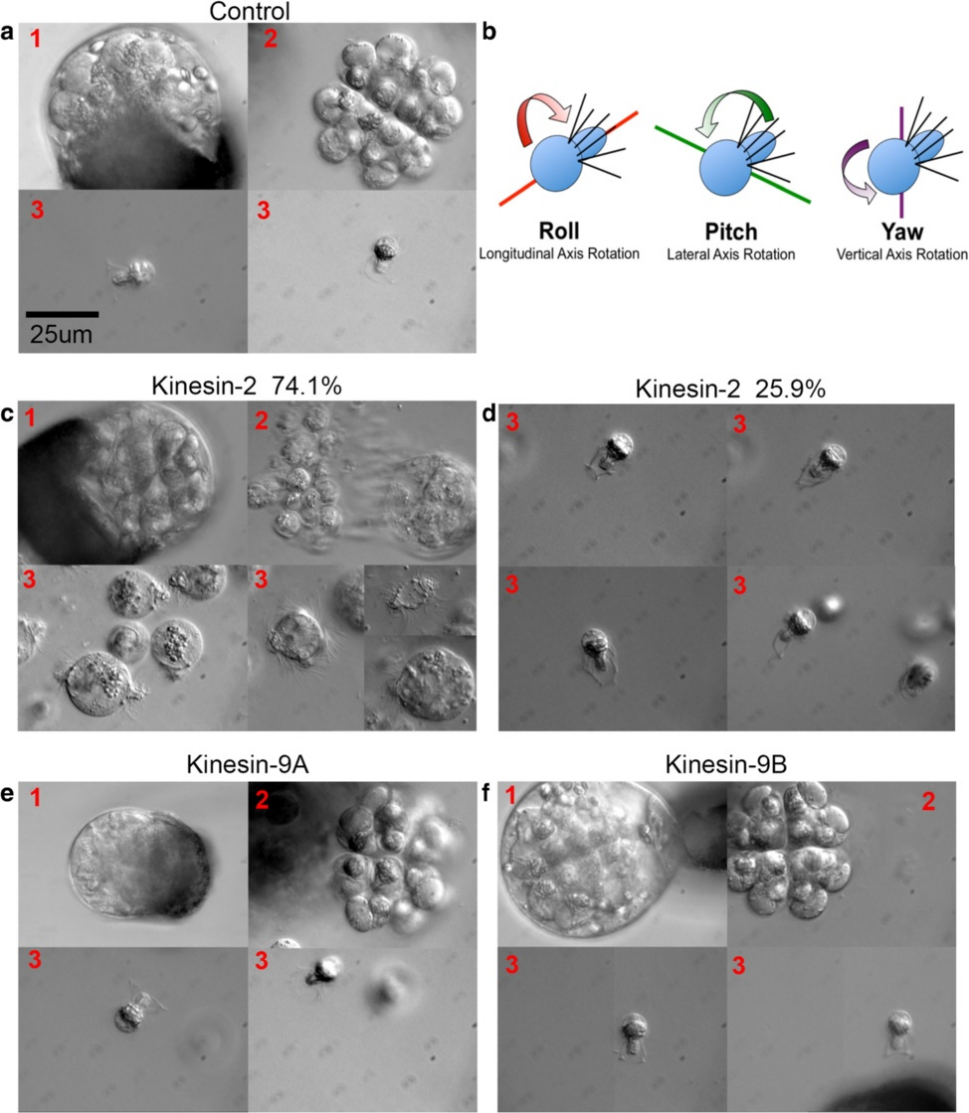
MvKinesin-2, MvKinesin-9A, and MvKinesin-9B have distinct roles in ciliogenesis and motility. **a** Untreated microspores during the final stages of spermatogenesis, visualized at 11 h. 1-Shedding of exine and thinning of intine around the microspore. 2-Release of 32 spermatogenous cells from each microspore in groups of 16 cells. 3-Motile spermatozoids emerge. **b** Cartoon representing spermatozoid swimming patterns. **c** MvKinesin-2 knockdowns visualized at 11 h. Thinning of the exine wall is normal, but cells emerge from the microspore as individuals. Spermatozoids have multiple sets of coils with attached cilia and are called *Monster*. **d** In a minority of MvKinesin-2 knockdowns spermatozoids appear to have longer cilia compared to controls and are called *Rapunzel*. MvKinesin-2 knockdowns quiver or roll (*Monster*) and swim in place (*Rapunzel*). **e** MvKinesin-9A knockdowns visualized at 11 h. Spermatozoids emerge and appear normal. MvKinesin-9A knockdowns pitch and yaw in place. **f** The microspore cell wall thins and spermatozoids emerge normally at 16 h of development in MvKinesin-9B knockdowns. Development and emergence is slowed relative to controls, and this phenocopy is termed *Late Bloomer*. Controls/MvKinesin-9B knockdowns roll in place and rapidly swim directionally

To determine whether the abnormalities observed with these kinesin knockdowns during spermatogenesis had any impact on ciliogenesis or on motility in released gametes, we observed the kinesin knockdowns at 11 h of development. At 11 h, the normal process of spermatogenesis reaches completion, and 32 motile spermatozoids, each with ~140 cilia, break free from their enclosing microspore walls. First, the opaque outer exine wall of the microspore begins to thin, revealing a much thinner and translucent intine wall (Fig. 4a–1). Then two clusters of 16 spermatozoids emerge from the microspore (Fig. 4a–2). The gametes are each contained within a thin wall-like structure built from an extension of the cytoplasm that originally surrounded the spermatid. Each motile spermatozoid rapidly spins in place, presumably in order to break free from its surrounding wall (Additional file 4). The shape of each spermatozoid resembles a corkscrew with cilia extending vertically from the edges of the microtubule and nuclear coil (Fig. 4a–3). Once released, the spermatozoids swim in a shallow helical path, rotating as they swim. By borrowing terms from aerodynamics, we have termed this rotational behavior “rolling” (Fig. 4b, Additional file 5). This pattern is repeated until the spermatozoid dies or reaches the egg cell in the female gametophyte (produced by the megaspore) for fertilization.

After the silencing of MvKinesin-2, for the majority of gametophytes (20/27; 74 %), the emergence of spermatozoids from the spore wall differed considerably from controls. Although the exine wall thinned as usual (Fig. 4c–1), the spermatozoids exited from the microspore as individuals, rather than as groups of 16 cells (Fig. 4c–2, Additional file 6). The spermatozoids that emerged this way were much larger than controls and often contained two or more coiled microtubule ribbons with attached cilia. Also, these cells lacked the characteristic extension of cytoplasm that unusually surrounds each spermatid (Fig. 4c–3). Due to their large size and multiple coils (Fig. 4c–3), these cells were unable to swim and could only quiver in place or yaw along their y-axes (Fig. 4b, Additional file 7). We call this phenocopy ‘*Monster*’ because each cell has multiple coils and cilia. It is likely that these cells failed to complete cytokinesis during the last spermatogenous cell division, but nevertheless, they were able to form microtubule coils in association with elongated nuclei and subsequently, each coil was associated with it complement of cilia.

In a minority (7/27; 26 %) of MvKinesin-2 knockdowns, the spermatids emerged from the microspore as normal; however, these spermatozoids exhibited abnormally long ciliary axonemes (Fig. 4d–3). The spermatozoids with long cilia were unable to swim in a shallow helical path. These cells could only roll and essentially, swim in place (Fig. 4b, Additional file 8). We refer to this phenocopy as ‘*Rapunzel*’ for its long cilia. *Monster* and *Rapunzel* are two different phenocopies caused by MvKinesin-2 knockdowns. It is possible that MvKinesin-2 participates in two distinct processes during spermatogenesis; the first occurs during cytokinesis leading to spermatid formation, and the second occurs during ciliary axonemal formation and affects ciliary length control. An alternate explanation is that the *Monster* and *Rapunzel* phenocopies are two manifestations of the same biological event caused by knockdown of kinesin-2. In this scenario, the *Monster* phenocopy is manifested earlier during spermatogenesis and *Rapunzel* later, in ciliogenesis. Because *Monster* spermatids were released from the microspores as groups of cells and *Rapunzel* spermatids as individuals, we suspect that each of these phenocopies occurs in different microspores and that all the spermatozoids emerging from one microspore display the same defects in development.

The thinning of the exine wall and the emergence of the spermatids from the microspore is normal in kinesin-9A knockdowns (Fig. 4e–1, 2). Once the spermatozoids emerge (Fig. 4e–3), their swimming behavior is affected by the knockdown (Additional file 9). These gametes roll, yaw, and pitch in place (Fig. 4b), effectively spinning without vectorial movement. Directional swimming for these cells occurs infrequently; they travel only short linear distances before switching back to spinning behavior (Additional file 9). Based on the movies, it is difficult to determine why the kinesin-9A knockdowns are unable to swim properly.

We have named our kinesin-9B knockdowns ‘*Late Bloomer*’ because the cells emerge slowly after the seemingly normal thinning of the exine (Fig. 4f–1, 2). The cells finally emerge ~16 h after microspore hydration, in contrast to 11 h for controls, and for the MvKinesin-2, and MvKinesin-9A knockdowns. The *Late Bloomer* spermatozoids that emerge are morphologically normal and have the same shape as control gametes (Fig. 4f–3). These cells also exhibit a helical swimming behavior similar to that of normal cells (Additional file 10).

### MvKinesin-9A knockdowns have irregularly positioned cilia

**Fig. 5:**
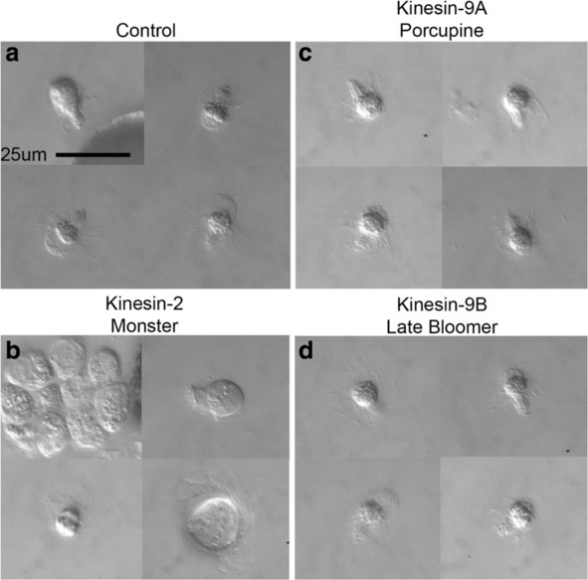
MvKinesin-9A knockdowns have irregularly positioned cilia. **a**–**d** After spermatozoids emerged from the microspore, they were fixed in 2 % PFA on a coverslip and visualized with DIC microscopy. **a** Untreated cells have a characteristic corkscrew shape and cilia can been seen emerging from the microtubule ribbon and organelle coil. Developing microspores treated with (**b**) MvKinesin-9A, (**c**) MvKinesin-2, and (**d**) MvKinesin-9B dsRNA. **b** Cilia that appear to emerge from all parts of the spermatozoid cell body, not just the coil. **c** Spermatozoids are larger than controls and do not have the characteristic corkscrew shape. **d** Spermatozoids appear normal and look similar to controls

To determine why kinesin-9A knockdowns were unable to swim normally when compared to controls, we used a variety of methods to observe the arrangement of cilia on the fully developed spermatozoids. Firstly, we fixed fully emerged spermatozoids quickly in 2 % PFA on the surface of a microscope slide. We then observed these fixed cells with DIC microscopy. In controls, the corkscrew shape of the spermatozoid cell body, the nuclear coil, and the ciliary axonemes can clearly be seen (Fig. 5a). Normal spermatozoids have the cilia placed in two regular rows along the dorsal flank of the anterior, coiled cell body [11, 43]. Kinesin-9A knockdowns show cilia that are irregularly positioned on the spermatozoid cell body and are not restricted to the anterior portion of the cell (Fig. 5c). We named this phenocopy ‘*Porcupine*’ for the cilia that emerge from all points of the cell. None of the other kinesin silencing treatments exhibited irregularly placed cilia similar to *Porcupine* in spite of the fact that MvKinesin-2 knockdowns (*Monster*) generally have cell bodies that are misshapen and larger than normal (Fig. 5b) and Kinesin-9B knockdowns (*Late Bloomer*) appear to be structurally normal even though they emerge at 16 h instead of 11 h after spore hydration (Fig. 5d).

### Cilia are irregularly positioned in MvKinesin-9A knockdowns due to the improper positioning of basal bodies in these cells

The abnormal distribution of the cilia and aberrant swimming patterns observed in the *Porcupine* phenocopy appears to be caused by the improper positioning of basal bodies along the microtubule ribbon in these cells (Fig. 3d). In order to test this hypothesis, we sectioned fixed microspores at 10.5 h after spore hydration and stained the sections with TBO, DAPI, and an anti-centrin antibody. Sections were visualized with phase contrast and fluorescence microscopy.

**Fig. 6:**
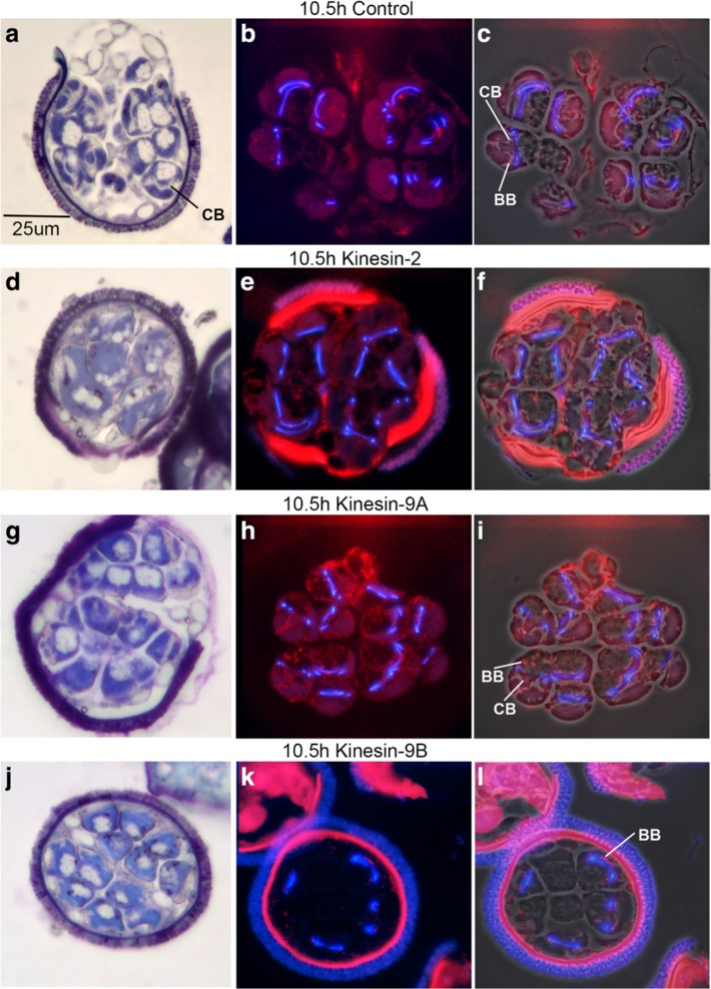
MvKinesin-9A is required to position of basal bodies during gametogenesis. **a**–**c** Untreated, control microspores developed for 10.5 h, embedded in methacrylate, sectioned, and stained with (**a**) TBO or (**b**–**c**) anti-centrin antibody (*red*) and DAPI (*blue*). **c** Fluorescence images are overlaid on phase contrast images of the same microspore. The organelle coil as well as the ciliary band (CB) is detectable in most spermatogenous cells. Anti-centrin labeled basal bodies (BB) are most prominent anterior to the nuclear coil in the area containing the ciliary band. Microspores treated with (**d**–**f**) MvKinesin-2, (**g**–**i**) MvKinesin-9A, or (**j**–**l**) MvKinesin-9B dsRNA and allowed to develop for 10.5 h before being embedded in methacrylate, sectioned, and stained with (**d**, **g**, **j**) TBO or (**e**, **f**, **h**, **i**, **k**, **l**) anti-centrin antibody and DAPI. **f**, **i**, **l** Fluorescence images are overlaid on phase contrast images of the same microspore. **d**–**f** Spermatogenous cells are larger and divisions between the cells are unclear. Centrin staining is diffuse although some centrin aggregates that resemble basal bodies can be found. **g**–**i** The ciliary band is at irregular angles to the nuclear coil and is not clearly defined. Basal bodies are not restricted to regions anterior to the nuclear coil but are randomly localized throughout each spermatid. **j**–**l** The nuclear coil and ciliary band are not defined. Basal bodies are apparent and are localized at regular intervals along the nuclear coil, similar to at 8 h of development

At 10.5 h of development, the normal spermatozoids are fully developed, but have yet to emerge entirely from the microspore wall. Using TBO staining at this time point, the organelle coil can easily be observed in each nearly-mature spermatozoid. Cilia emerging along this coil are also visible. The cilia are wrapped around the cell body coil and are seen in transverse-section in some of the gametes as a white area adjacent to the organelle coil. We refer to this area as the ciliary band (Fig. 6a). Anti-centrin staining in control sections form aggregates that resemble basal bodies. In control spermatids, the basal bodies are restricted to areas on the dorsal side of the coiled nucleus (Fig. 6b). Overlaying the fluorescence images with phase contrast images of the same section, it appears that the centrin aggregates associate uniformly along the edges of the organelle coil and are often within areas of the ciliary band (Fig. 6c).

In comparison with untreated gametophytes, in MvKinesin-9A knockdowns stained with TBO, it is difficult to visualize the ciliary band. In cells that possess a visible ciliary band, the band is irregularly shaped compared to controls (Fig. 6g). This suggests anomalies in the orientation and localization of the basal bodies in the spermatid. Centrin staining confirms this, showing aggregates that resemble basal bodies present throughout the cytoplasm of the nearly-mature spermatozoids. Aggregates of centrin are present at the dorsal face of the spermatozoid along the edges of the organelle coil as in controls, but they are also abundant along the posterior portion of many spermatozoids (Fig. 6h, i). The failure of these cells to position the axonemes correctly during differentiation is a likely explanation for the *Porcupine* phenocopy and the odd swimming patterns observed in these knockdowns.

In contrast to this phenocopy, MvKinesin-2 knockdowns visualized with TBO have large, irregularly shaped cells. We suspect this is the consequence of arrested, aberrant or blocked cell division cycles. Coils are visible only in some of the maturing spermatids (Fig. 6d). Anti-centrin antibody staining in these knockdowns appears as a diffuse cloud of fluorescence in spermatogenous cells. Aggregates that resemble basal bodies are present in some cells; however, they do not appear localized in any reliable pattern that resembles basal body distributions in normal cells (Fig. 6e, f). This pattern is reminiscent of the *Monster* phenocopy where the released gametes are larger than normal and possess multiple coils containing cilia.

In kinesin-9B knockdowns, the spermatogenous cells resemble to control cells, though the coil and cilia are not as apparent (Fig. 6j). Anti-centrin antibody staining forms aggregates that resemble basal bodies and are positioned at regular intervals along the anterior portion of the nuclear coil (Fig. 6k, i). These cells do not look like they have developed to the same stage as the controls though clearly, they have proceeded into the later stages of differentiation. This provides further evidence that knockdown of kinesin-9B (*Late Bloomer*) causes a general slowing of differentiation.

## Discussion

### MvKinesin-2 has atypical functions in cytokinesis and ciliogenesis during male gametophyte development in *Marsilea*

Phylogenetic analysis of MvKinesin-2 shows that this kinesin is atypical when compared to most other members of the kinesin-2 family. Firstly, MvKinesin-2 cannot be placed in the usual kinesin-2α, β, or γ subgroups, though it most resembles the kinesin-2 isoform found in *Physcomitrella*. This was unsurprising since both FLA10 and FLA8 in *Chlamydomonas* [14] and the kinesin-2 in *Physcomitrella* also group independently (Fig. 1a). Similarly, many non-animal kinesin-2 s also cannot be placed in the established kinesin-2α, β, or γ subgroups (Fig. 1a). Secondly, we found only a single kinesin-2 transcript in our transcriptome. This was surprising because kinesin-2 is best known to function in heterotrimeric complexes. A single kinesin-2 motor can also be found in *Physcomitrella* and in certain protists [35–37]. Although the reason is unclear, organisms that are only ciliated for a short time during the life cycle show a general reduction in cilia-associated proteins [35]. The single kinesin-2 motor found in *Marsilea* may function as a homodimer, similarly to *C. elegans* OSM-3, though evidence to support this existence of homodimeric kinesin-2 outside of sensory cilia is limited [44]. From this analysis, we were unable to discern whether in gametophytes MvKinesin-2 functions as a homodimer, with yet unidentified partners, or if it acts alone. It is possible that MvKinesin-2 and single kinesin-2 proteins found in other organisms function separately from the more well studied kinesin-2 motors.

Functional silencing assays showed that MvKinesin-2 is required for cytokinesis during the cell divisions that ultimately produce 32 spermatids in the gametophyte (*Monster* phenocopy) and for regulating the length of cilia (*Rapunzel* phenocopy). However, we cannot eliminate the possibility that the *Monster* and *Rapunzel* phenocopies represent two different manifestations of the same biological event where the role for MvKinesin-2 in cytokinesis precedes its involvement in ciliogenesis. *Monster* spermatids formed can still produce motile cilia, but these abnormally large gametes contain multiple coils with attached cilia and are unable to swim and can only quiver in place (Additional file 7). In addition, these cells lack the extension of cytoplasm that usually surrounds the anterior portion of each spermatid (Fig. 4c–3). Although not typically associated with kinesin-2, a role for this motor in cytokinesis has been reported [45–47]. How exactly kinesin-2 participates in cell division has yet to be established as mutants are able to pass through mitosis without visible consequence [48–50]. We suspect that the cytokinesis defect phenocopy observed in *Marsilea* is related to the function of kinesin-2 in the intracellular transport of Golgi-derived vesicles and membrane dynamics [45, 51–53]. During plant cell cytokinesis, the coordinated efforts of a variety of motor proteins are necessary to deliver Golgi-derived vesicles along phragmoplast microtubules to build the cell plate [54]. Heretofore, kinesin-2 has not been implicated in this process and a recent study in *Physcomitrella* showed that kinesin-2 was not expressed in caulonemal cells undergoing mitosis [55]. Since both *Marsilea* and *Physcomitrella* only produce ciliated cells in their gametophytes, it is likely that the expression of kinesin-2 is restricted in land plants to the formation of male gametes. It is possible that this motor has evolved or retained a role in cytokinesis and membrane dynamics specifically for spermatogenesis.

In a minority of cells, MvKinesin-2 knockdowns produced abnormally long ciliary axonemes (*Rapunzel* phenocopy) while maintaining normal cell size and shape. This result suggests a function for MvKinesin-2 during ciliogenesis that is distinct from its role in cytokinesis. *Rapunzel* spermatozoids were unable to swim vectorially and could only rotate in place. Heterotrimeric kinesin-2 is responsible for anterograde transport during ciliogenesis. Mutations in this kinesin isoform typically result in no change in ciliary length or the absence of full-length cilia due to suppressed IFT activity [15, 17, 21, 22, 56]. Therefore, the production of long cilia was unexpected. One possible explanation is that other proteins are responsible for modulating IFT dynamics and ciliary length during male gametophyte development in *Marsilea*. Evidence in support of this hypothesis can be found from the cephalic male (CEM) cilia of *C. elegans*. Here loss of KLP-11 (a member of the heterotrimeric kinesin-2 complex) results in long cilia due to the complex interaction of KLP-11, OSM-3, and the kinesin-3 motor, KLP-6, during IFT [57]. In *Chlamydomonas* kinesin-13 is important for regulating axoneme assembly and length [58, 59] and KCBP, a plant-specific member of the kinesin-14 family, is necessary for the function of flagella [60]. It is possible that kinesin-13, KCBP, other plant-specific, or orphan kinesins are responsible for modulating IFT dynamics during ciliogenesis in *Marsilea*. Additional functional studies on these kinesins are necessary to investigate this possibility.

### Kinesin-9 is required to orient basal bodies along the microtubule ribbon and for spermatid differentiation during spermiogenesis

Our analysis of the kinesin-9 family echoes previous findings that there are two kinesin-9 subgroups [28, 29]. In the *Marsilea* gametophyte transcriptome, a transcript that encodes one member of each kinesin-9 subgroup is present. With silencing treatments, we found that kinesin-9A is necessary for the organization and placement of basal bodies that become the sites of ciliogenesis during spermatid maturation. Since the basal bodies are not properly placed along the microtubule ribbon, and, as a consequence, the cilia are misplaced in the cell body, the spermatozoids cannot maintain normal swimming patterns and they are only able to spin and flip in place. In both *Chlamydomonas* and *T. brucei*, kinesin-9A is also important for motility; however, this is achieved through the interaction of kinesin-9A with central pair microtubules in *Chlamydomonas* [29, 31] and not through regulating the orientation of basal bodies. The typical phenotype observed in kinesin-9A mutants of a reduction in ciliary beating [29, 31] was not observed in *Marsilea* kinesin-9A knockdowns. Instead, cilia were unable to maintain normal swimming and the gamete moved in a highly disorganized pattern (Additional file 9).

We also found that knockdowns of kinesin-9B produced a general slowing of differentiation as fully mature and functional spermatozoids were released 5 h later than controls. Early stages of development were not altered after kinesin-9B knockdown. This result implies that kinesin-9B is needed for the differentiation of spermatids into motile cells. The specific role for kinesin-9B in differentiation is not known, but we suspect that there is sufficient redundancy among kinesin isoforms to allow development to proceed, albeit slowly, in the absence of this motor protein. Functional studies on kinesin-9B in other systems have begun to appear in the literature. In *T. brucei*, kinesin-9B is responsible for building the paraflagellar rod (PFR) [29]. The PFR is unique to kinetoplastic protozoa like *T. brucei* so, in a fashion similar to the spermatozoid of *Marsilea*, it is not surprising in other eukaryotes that kinesin-9B would perform functions beyond PFR assembly during ciliogenesis.

## Conclusions

Among the 56 kinesin family members whose mRNAs are detectable in the male gametophyte of *Marsilea* [38], we have found that kinesin-2, kinesin-9A, and kinesin-9B are each required for specific events during spermatid formation or maturation and for ciliary assembly or positioning during rapid development. mRNAs that encode these kinesins increase in abundance during the developmental stage associated with spermatid differentiation and ciliogenesis. RNAi knockdowns of each of these kinesins show distinct and separate phenocopies during spermatogenesis and spermiogenesis. Functional analyses showed that kinesin-2 is involved in cell division (*Monster* phenocopy) and in regulating the length of motile cilia (*Rapunzel* phenocopy). Knockdown experiments showed that kinesin-9A is required for the proper positioning of basal bodies (*Porcupine* phenocopy) during spermatid differentiation leading to spermatozoids that are unable to power directional swimming. In contrast, kinesin-9B (*Late Bloomer* phenocopy) does not appear to be required for the construction of motile spermatozoids in *Marsilea*.

Heterotrimeric kinesin-2 is the main motor associated with anterograde IFT in motile cilia and mutations typically produce cells that lack cilia due to resultant problems with IFT [15–18]. Therefore, the involvement of kinesin-2 in cytokinesis and in regulating ciliary length in the *Marsilea* male gametophyte is atypical. Although best known for its functions during ciliogenesis, kinesin-2 has also been implicated in mitosis and in intracellular membrane trafficking in a number of cell types [45, 51–53] thus potentially explaining the *Monster* phenocopy observed during gametophyte development. Also kinesin-2 is not the only motor implicated in anterograde IFT. In the *C. elegans* cephalic male cilia (CEM) KLP-6, a member of the kinesin-3 family, works together with kinesin-2 during IFT to regulate ciliary length [57]. It is possible that the *Marsilea* kinesin-2 interacts with other motors during ciliogenesis and in IFT thus explaining the *Rapunzel* phenocopy. The Porcupine phenocopy observed in Marsilea after knockdown of kinesin-9A was also different than currently established roles for this motor. The typical phenotype observed in kinesin-9A mutants of a reduction in ciliary beating due to the interaction of kinesin-9A with central pair microtubules [29–31] In *Marsilea*, we attribute disorganized ciliary beating and the inability to maintain normal swimming patterns on the mislocalization of basal bodies in mature spermatozoids. The reason for these contradictions is not currently understood; however, significant differences in the structure of axoneme in fern spermatozoids including those from *Marsilea* [61–63] may be to blame.

## Methods

### Sporophytes of *Marsilea vestita*

Our original collection of sporocarps of *M. vestita* was obtained from Dr. Peter K. Hepler (University of Massachusetts), who had initially collected sporocarps in the 1970s from Lake Lagunita, which is located on the Stanford University campus in California. Dry sporocarps remain viable for extended periods [1, 42]. In our annual effort to raise new sporophytes, approximately 20–30 dry sporocarps were scarified and immersed in water in an aerated aquarium to activate development in microspores and megaspores. After 1 or 2 weeks, small sporophytes were transferred to water-saturated soil in growth flats and raised indoors until they were approximately 1–2 cm in height. At this stage they were transferred to pots and placed into artificial ponds located at the University of Maryland Research Greenhouse Complex, where they were allowed to grow for 6–8 months as semi-submersed plants [42]. By this time, the plants were actively making new sporocarps. Thereafter, we allowed the ponds to dry down gradually over the course of 2–3 months and during this period, the sporophytes turned yellow, and the spores contained in sporangia within the sporocarps underwent their natural desiccation process. Sporocarps were then harvested from the dried plant material and stored at room temperature. Successive plantings over the years allowed us to create a stockpile of sporocarps that contain viable, desiccated spores. A set of sporophyte voucher specimens is being prepared for storage at the Norton-Brown Herbarium at the University of Maryland (College Park, MD).

### Culturing Gametophytes

*Marsilea vestita* microspores were isolated from dry sporocarps as previously described [1, 42]. Four milligrams of microspores were then cultured in 1 ml commercial spring water (Deer Park) for 1 h at 20 °C with rotational shaking. Gametophytes were then transferred to 50 ml flasks containing a total of 10 ml spring water and cultured with rotational shaking at 20 °C [42, 64].

### Identifying Kinesin-2 and Kinesin-9 Sequences in *Marsilea*

Kinesin-2 and kinesin-9 sequences were identified by searching the entire gametophyte transcriptome with kinesin-2 and kinesin-9 sequences from *Physcomitrella* (Phypa_425592, Phypa_425498, Phypa_458410, Phypa_428375) and *Chlamydomonas* (CrFLA8, CrFLA10, CrKLP1, CrKIF9b). Stand-alone BLAST was used for the analysis. A maximum e-value of 1E^-100^ was used to yield the best possible matches and avoid identifying kinesins unrelated to kinesin-2 and -9 in the search (Additional file 1).

### MvKinesin-2 and MvKinesin-9 Phylogenetics

Sequences for previously analyzed kinesin-2 and kinesin-9 family members were obtained using NCBI [65], Phytozome [66], and Cosmoss [67] databases. The motor domain from each sequence was identified using the NCBI Conserved Domain Database [68] and isolated for analysis. Phylogenetic trees for kinesin-2 and kinesin-9 were built by importing the sequence files into MEGA [69] and generating a multiple sequence alignment using the ClustalW default parameters. Maximum likelihood (ML) phylogenetic trees were built using the GARLI (Genetic Algorithm for Rapid Likelihood Inference) web service [70, 71]. Trees were generated using a fast ML stepwise-addition algorithm and 50 attachment braches were evaluated for each taxon. The parameters used were as follows: rate matrix-LG, state frequencies-estimate, proportion of invariable sites-estimate, rate heterogeneity model-gamma distribution, number of rate categories-4. Bootstrap analysis was done at 500 replicates. Phylogenetic trees were then viewed using FigTree (Fig. 1a–b) [72].

### Poly(A) + RNA Isolation

Four milligrams of microspores were cultured in spring water for the desired amount of time. RNA was isolated from the developing gametophytes using the Magnetic mRNA Isolation Kit from NEB (#S1550S, New England Biolabs) according to manufacturer instructions.

### Primers

Primers for MvKinesin-2, MvKinesin-9A, MvKinesin-9B, and MvCentrin were generated using sequence data from the gametophyte transcriptome (Additional file 11).

### Reverse Transcription Polymerase Chain Reaction (RT-PCR)

Reverse transcription reactions were performed using poly(A+)-RNA isolated at 1–2 h, 3–5 h, and 6–8 h of gametophyte development. AMV RT enzyme (New England Biolabs) was used to catalyze the reaction according to manufacture instructions and 5 ng of polyA + RNA was used for each reaction. PCR was carried out using 10 μl of cDNA from each RT reaction and amplified using *Taq* polymerase (New England Biolabs) for 30 cycles. RT-PCR products were run on a 1 % TAE agarose gel and visualized using ethidium bromide and UV light.

### RNAi

dsRNA was constructed and RNAi was performed as described previously [64]. Spectrophotometry and gel electrophoresis was used to determine the quality and quantity of dsRNA used for RNAi (Fig. 2b). dsRNA construct sequences can be found in Additional file 11. RNAi was performed by adding 200 μg dsRNA to the microspores with 1 ml of spring water in a 2 ml microcentrifuge tube [42, 64]. After 1 h, gametophytes were transferred to 50 ml flasks containing a total of 10 ml spring water and cultured with rotational shaking at 20 °C.

### Fixing, embedding, and sectioning microspores

At the time of analysis, gametophytes were fixed with 4 % PFA, embedded in methacrylate, and sectioned as previously described [1, 42].

### Staining and Immunofluorescence

Toluidine Blue O (TBO) staining was performed on fixed and sectioned microspores and observed with bright field microscopy as previously described [73]. Immunolabeling of sectioned microspores was preformed as previously described [74, 75]. The primary antibody used was mouse anti-centrin clone 20H5 (Milipore 04-1624) diluted to 1:200 in PBS. The secondary antibody was Alexa Fluor 594-conjugated goat anti-mouse (Molecular probes cat# A11005) diluted to 1:1000 in PBST. DAPI was added at 2.5 μg/ml during the last 10 min of staining [75]. Incident light fluorescence imaging was conducted on a Zeiss Axio microscope with standard Fluorescein, TexasRed, and UV filter sets. For all images, thousands of microspores are treated, sectioned, and viewed. About 100 microspores were imaged for each treatment. Percentages were calculated by counting the incidence of each phenocopy.

### Analyzing Spermatozoid Swimming Behavior

Gametophytes were allowed to develop for 10.5–16 h and viewed by placing several drops of culture media containing emerging and fully developed spermatozoids in a shallow dish with a coverslip for a bottom. Spermatozoids were analyzed using differential interference contrast (DIC) microscopy with a Zeiss Axiovert 135 TV microscope. Movies were taken with a PIKE F032B monochrome camera (Allied Vision Technology) using StreamPix software at 30 frames per second. Movie files were converted to .mov format. Still frames from movies were captured and converted into tiff format for analysis.

### Visualizing Whole Spermatozoids

Spermatozoids were viewed by transferring a drop of the culture media at 11 h to a glass microscope slide. A drop of 4 % PFA was added to fix the spermatozoids rapidly. A coverslip was then placed on top of the drop containing spermatozoids, culture media, and PFA. To prevent crushing fully developed spermatozoids on the slide, the edges of the coverslip were coated in a thin layer of Vaseline. Spermatozoids were then visualized using DIC microscopy.

## Abbreviations

ATP, adenosine triphosphate; B, blepharoplast; BB, basal bodies; Blastp, basic local alignment sequence tool, protein; CEM, cephalic male cilia; Cr, Chlamydomonas reinhardtii; DAPI, 4’, 6-diamidino-2-phenylindole; DIC, differential interference contrast microscopy; dsRNA, double stranded RNA; e-value, expectancy value; FLA8, flagellar assembly protein 8; FLA10, flagellar assembly protein 10; GARLI, genetic algorithm for rapid likelihood inference; IFT, intraflagellar transport; J, jacket cells; KAP, kinesin associated protein; KCBP, kinesin calmodulin binding protein; KIF, kinesin family member; KLP, kinesin-like protein; mRNA, messenger RNA; ML, Maximum likelihood; Mv, *Marsilea vestita*; OSM-3, osmotic avoidance abnormal protein 3; PFA, paraformaldehyde; PFR, paraflagellar rod; Poly(A), polyadenylated; RNA, ribonucleic acid; RNAi, RNA interference; RNAseq, RNA sequencing; RT-PCR, reverse transcriptase polymerase chain reaction; SP, spermatogenous cells; Tb, Trypanosoma brucei; TBO, toluidine blue-o

## Additional files

Additional file 1: Identifying kinesin-2 and kinesin-9 sequences in *Marsilea*. (A) Protein sequences from members of the kinesin-2 and kinesin-9 family in *Physcomitrella patens* and *Chlamydomonas reinhardtii* were used to search the *Marsilea* transcriptome. (B) Stand alone blast used to search the transcriptome for kinesin-2 and kinesin-9 sequences. Results show that there is one kinesin-2 mRNA sequence (KT986235) and two kinesin-9 mRNA sequences (KT986258, KT986259) in the male gametophyte of *Marsilea*. (*PDF 3038 kb*) Additional file 2: Building a kinesin-2 and a kinesin-9 phylogenetic tree. Source of kinesin-2 and kinesin-9 motor domains used for multiple sequence alignment (MSA) and to build phylogenetic trees. (PDF 1418 kb) Additional file 3: Domain architecture and multiple sequence alignments for (A) kinesin-2, (B) kinesin-9A, and (C) kinesin-9B. Bold regions correspond to the motor domains of each respective kinesin. Regions that are important for motor domain function, including P-loop (yellow), switch I (blue), and switch II (green), are represented in each motor. Regions that are within the motor domain are highlighted and are very highly conserved. Non-motor regions are somewhat conserved, however, large gaps and insertions exist in each alignment. Alignments generated using T-coffee [76, 77]. (PDF 1034 kb) Additional file 4: Video 1. Untreated, control spermatozoids emerge from the microspore. (MOV 4129 kb) Additional file 5: Video 2. Untreated, control spermatozoids exhibit normal swimming behavior by spinning in place. (MOV 3274 kb) Additional file 6: Video 3. MvKinesin-2 knockdowns emerging from the microspore. (MOV 5165 kb) Additional file 7: Video 4. MvKinesin-2 knockdowns swimming in place exhibiting multiple coils with attached cilia on one cell body (*Monster*). (MOV 4106 kb) Additional file 8: Video 5. MvKinesin-2 knockdowns swimming in place exhibiting long cilia (*Rapunzel*). (MOV 4527 kb) Additional file 9: Video 6. MvKinesin-9A knockdowns swimming erratically. (MOV 4190 kb) Additional file 10: Video 7. MvKinesin-9B knockdowns swimming in a similar manner to control spermatozoids (*Late Bloomer*). (MOV 4043 kb) Additional file 11: Primers and dsRNA constructs. Primers for MvKinesin-2, MvKinesin-9A, MvKinesin-9B, and MvCentrin were generated from the gametophyte transcriptome for (A) RT-PCR and (B) to make dsRNA. (C) Full dsRNA constructs for RNAi. (PDF 564 kb)
