# Supplementary material for: Kinesin-2 and kinesin-9 have atypical functions during ciliogenesis in the male gametophyte of Marsilea vestita
Source: BMC Cell Biol. 2016 Jul 16;17:29. doi: 10.1186/s12860-016-0107-7 (PMC4947347; doi:10.1186/s12860-016-0107-7)
Supplement: Additional file 1: — Identifying kinesin-2 and kinesin-9 sequences in Marsilea. (A) Protein sequences from members of the kinesin-2 and kinesin-9 family in Physcomitrella patens and Chlamydomonas reinhardtii were used to search the Marsilea transcriptome. (B) Stand alone blast used to search the transcriptome for kinesin-2 and kinesin-9 sequences. Results show that there is one kinesin-2 mRNA sequence (KT986235) and two kinesin-9 mRNA sequences (KT986258, KT986259) in the male gametophyte of Marsilea. (PDF 3038 kb) [file 12860_2016_107_MOESM1_ESM.pdf]

A

>XP_001697037 (Cr_FLA8)
MASGGECVKVAVRCRPLNGKEKGDNRATIVEVDNKTGQVTLNNPKGDEPPKTFTFDNAFDWNVTQRDVYDVVARPIVNSVMDGYNGTIFAYGQTGTGKTHTMEGFP
TPELQGIIPNCFDHVFETVNSSTGKQWMVRASYLEIYNEEVRDLLSKDPKNKLELKEHKDSGVYVKGLNAFVVKGVPELKNVLEVGKKNRSVGATLMNQDSSRSHSIFT
ITIETIEQTQAQPEGHIRVGKLNLVDLAGSERQSKTGATGDRLKEATKINLSLSALGNVISALVDGKSGHVPYRDSKLTRLLQDSLGGNTKTIMCANMGPADWNYDETLS
TLRYANRAKNIKNKPKINEDPKDAMLREFQDEIARLKAALEAEGGALPEGFATGPGGEIIVEKVVQVPKALDASFLEQMRKDMEEQMKKELASQQAAALNDEQLQKVK
EEAAAKAKAEAARLEEEKKKAEEEAARMQRKQQKIKAEMDKKSLDAEQIRAEKEALAKKLKAMESKILKGDQAGGLAEVTKKKEEELKRKEQELERRRKEEEEQRKK
IQVMEEQQLAMEDKYKDKADEADQKTKKLKKLWKKFQEVNAEVEDMYKEFQREKEDLLESIRMLQDQMQLKDMVIEAFIPPEEVQKVMKRAHWDDEREVWVLERL
SDIGKRETAQGASRRPVSASGQRRPTSDFAKLANAMGDMNPRFKSENILNLELDLPERTTYDYEGPGVDPRVQAAINAAFAEDGELIFVGSEQNVHLGDASAAAARPDS
AKKRPASARKGTKK

>XP_001701510 (CrFLA10)
MPPAGGGSESVKVVVRCRPLNGKEKADGRSRIVDMDVDAGQVKVRNPKADASEPPKAFTFDQVYDWNCQQRDVFDITARPLIDSCIEGYNGTIFAYGQTGTGKSHTME
GKDEPPELRGLIPNTFRYVFEIIARDSGTKEFLVRSSYLEIYNEEVRDLLGKDHSKKMELKESPDRGVYVKDLSQFVCKNYEEMNKVLLAGKDNRQVGATLMNQDSSRS
HSIFTITIECIEKLESAAAQKPGAKKDDSNHVRVGKLNLVDLAGSERQDKTGATGDRLKEGIKINLSLTALGNVISALVDGKSGHIPYRDSKLTRLLQDSLGGNTKTVMVA
NIGPADWNYDETMSTLRYANRAKNIQNKPKINEDPKDAMLRQFQEEIKKLKEQLAARAAGGGGPITMPSGGGSPTQKIVERTEEVDPDIDAIKAQMRAELEAKMKSDIS
TEALDKAREEAEAAAKKQLQAIIDDQGKTEAQKKAARDALKKQAEEARAIAGAIEKEKQEKAVLESRIKEMEGKIVVGGVNMLEKVDELKQKSEDIKREAAIRKRQEE
EAKRRLEELQAAQVDADAKFASLDEEINVKSRQLKKLFEKYQGKKGELADLQEQFQREREGMLEDYRILTQQIKLKNLIIACFIPPDYQDKIMQHCHWQDYDSSWNIDCI
AYAGNAVRTNQELQAQEDKEHDAAAENERLKNCFFSYEQFEAAGAGSKQGGGGGGGGGGAARPGSSAGRAVGSAARRTGGKAGGKDITDIGSLRDSVNWGDDDDK
KKGAIPKAKGLVKDTVDPRLRASKLK

>Phypa_425592 (PpKinesin-2)
MKERGRGHGSGLTRTSSTGSSLSSGGGSARRAERVQVVVRCRPMLVKENAEGRNNCVLVDTVGSTIQVKNLKQPEQEPKLFTFDKTYDATSTQKQLYDDVAHPIVHSV
MCGYNGTVLAYGQTASGKTFTMDGLDDPPEMRGIIPQAFEGIFTHIQDSQSSDNFLVRASYLEIHNEEIRDLLATGSQSSSRLELKENVEGNVYVKNLTSITVQSVADISHL
LTVGKKSRSVGATLMNQDSSRSHSIFTITVEASARSSSAETDGSMHIRVGKLNLVDLAGSERLNKTGATGDRFRELTNINWSLSALGNVISALVDDKSSHVPYRDSKLTR
LLQDSLGGNTRTVMIANIGPADYNYDESVSTLRYANRAKSIKNKPRINEDPKDAILREFQEEIARLRAQLQASSPVLDERTPNKIDPSSPSAVDLELHMQQLRAKMQEDM
EHQVFLHKSMSDQAMACIKADFEQKTQSEMQALKAEKERSDEEKQRIAQQLQQQHVELQSHYQALAREKEDRDVLAAKLRALEEKLVHGTNNDNEKLLEKAKQKEV
ELALREQQLQEKENMDEERQRKIAELEEAQLMAEEKCNTMEEEVEMKTRKLRKLMARYQQSKNDINALRTELQDTIHEFQRERADMFLSLRSLDQQLQLKNFLIDKFIS
PEDLTKVMRRVHWDEENEIWNLVQNAPVRSLPYNGPTRVSIDGFTKQPLLRPNSAIGRSRPPLRQPEQTLSQSERDRYLSNTSSVHVHSPRKGAGRSKAIDEGKIGCRVD
NILKKDQDVSEGRNHFEFGDNSQLSSSKVDIEHALREFRVDNKPSFTWDREPQQVTPAAAGARPKSARTKQHNPRPRTAR

>Phypa_425498 (Pp_Kinesin9A)
MPYVDDALLSRIRVYLRLRPSVKPSPAINIESETHRVLIDVEKSIGGGPPKAYVNQIVFNVDNIVQVYVHRGIIPRAIQQIFEEKEAKPEAGIVVHMSYMEIYQEGLYDLLQ
KRRDDLMIIEDNQLLNVRGLAKVRVETETEALKWFQEGEKSRSFGNHFLNSLSSRSHTILTFYMERRVARVSTQLALQVAKLNLVDLAGVERLKKTKGDTGSLMRKEA
CINNKTLSFLEQTIFALRLKKAHIPFRHSKVTTLLKESLGNNHKTVFMVCAWPEEYFLDETIGALRFAQRVKYLKIFQVTHKKPDCADTTRKQQLEIATLKQELALKDAL
NGRPGLGYDNLT

>Phypa_458410 (Pp_Kinesin9A)
MPSGEPLSSIGIKVHVRIRPTAKPSPMFHIEDQTNTVLIDLLKQNGGGAPESFVDQIAFSVNSITQSTDQSEVYESCGRALVKDFLEGYNATFIAYGQVGSGKTYTMAGDM
KVNAHRGFIARAIHQIFEEKEADPGSGIVLYISYLEIYQERSKDLMIIEESGYVYIRGLAKIPVETEAQALMCFSEGEKQRSYACHQINQVSSRSHTIFTLCMEKRVGRFKTE
YDTVVAKLNLVDLAGFERLKKTNTSTGGRMRVEACSINKSLCLLEQAVYAIKQGQEYVPFRQSKISIILKEALSGNCRTELILCLWPEEYFLDETVRRQNLKTHSRKYFVR
HAQEIANLQQELALKDALQGRPGLSFDDLTLENYSLYSRSLKYLCELK

>Phypa_428375 (Pp_Kinesin9B)
MQLFVSSTKFSAMGAGFDSTIDIYLRVRPISSGAKAVLELNQEEGRVTWTIPRHVSLGLANHQREHFTFKFTGLFDMESKQDEVFQKVAHKVVIGSLDGYNGTIFAYGQT
GSGKTYTITGGSERYVDRGIIPRTISLIFSEIAERSEYAYTLHFSYMEVYNETGYDLLNPDHETKALEDLPKDFILANEPIIANYQFANAFRVATNPDVHFLQNPAGLGRNR
WNSNEEEALNLVFVGDTNRIISSTPMNMASSRSHCIFTAHILACKVGEETVRKSKLHLVDLAGSERVWKTGVDGQVIVALQEKFQGKMRTHIPYRNSMMTSVLRDSIGG
NCLTVMIATVTIAQDQLPETISTCRFAQRVAMISNQVTLNEEVDPNLLIKRLKQQIGDLKGEIALLRGENENRPPLSASEIENVRSRVIGFLSDKGVDTDLHCGGSMMHIHA
AFQILKEMLKHGETQTIKSVQDDNGSQVSSESTVVKTLQVKVSDLQYQLQQRDNEIQILVAFIRKREAAARNTVRSASVQSLTDRPPSTSCRGSRTEEKGGEMKRSQLLA
EKNVTPRPSKSADFHQPKSTELDVDLGVAKLPVTSGREMDKDKAFELFSKGNDKMEAIEENKSLLIAMCANAKALGEKVNRARDRTYKEGFSELRDIKKEIEHLHKLLE
HNGAQLQADFEVYWSAMTAAPASTETSRKAPIIPRLQISSAIGPQHQANQSSSAEYKPLFPFSSNSLSARGSHCSPLSVSDPRSGSRGGNDLRALALASSKEHSKYVKSPSS
SGAGQVLNNLDNLLQPIRRQQLKESVNTAGLQKVPLTGNAAADADIIAFYKARSGVLKKVSS

>XP_001701617 (Cr_KLP1)
MVKQAVKVFVRTRPTATSGSGLKLGPDGQSVSVNVPKDLSAGPVNNQQEQFSFKFDGVLENVSQEAAYTTLAHEVVDSLMAGYNGTIFAYGQTGAGKTFTMSGGGTA
YAHRGLIPRAIHHVFREVDMRADKMYRVHVSYLEIYNEQLYDLLGDTPGTSDALAVLEDSNSNTYVRGLTLVPVRSEEEALAQFFLGEQGRTTAGHVLNAESSRSHTVF
TIHVEMRTSDAASERAVLSKLNLVDLAGSERTKKTGVTGQTLKEAQFINRSLSFLEQTVNALSRKDTYVPFRQTKLTAVLRDALGGNCKTVMVANIWAEPSHNEETLST
LRFASRVRTLTTDLALNESNDPALLLRRYERQIKELKAELAMRDTLSGKGRVSYDDLTDDELRELHATCRRFLHGEAEPEDLPADSMKRVRETFKALRAVHVAIKADM
ATQMATLRRATEEGSGAAARGGDSAGPSGVGDVDLRATGGFTVGHAPLDARPPVRSELGSPGAGASGAEALGEPRSPGGGLHAQASSHTDAGSNWGDAGPLSSPGGT
RLAGIFGVSGDRNAVFRRYKVDVGEGRELAASLKAASIALADTKASIRSLGASVNDAKQRIDELSSALALRRGATPAGGDGEVLDSEAYALMQELKSAKSRYRTDFDSL
KSAREELEPQIQAVAVARAGLLEAFDRWAAAQSDTTLKRMATAGRAMSGIAPGEEDEMDAGEQFERMQIARISERDPDSLAFHTALKRTGAAVSRPATVATGGNAKA
AAMATRKMEHTQAVNRGLAR

>**Cre01.g036800.t1.1 (Cr_Kinesin9B)**
MAPGPEVAGIDIFVRVKPVPKPSPRLGIDNSENKVEFNIPRNEAAGLVNNQREHFEFRFNGILQADAKQDEVFERVARPVVMGAMEGYNGTIFAYGQTGSGKTFTITGGP
ERYVDRGIIPRSISAIFSEISKRHDYQYSVHISYLEIYNNEGYDLLDAEREIKALEDLQQVHVGEGEDGTVSYRNLSMYRANNEEEALNLLFLGDTNRTISETPMNQASSRS
HCIFTIHVEARKTGEDVVRRSKLNLVDLAGSERVSKTGVDGTTLREAKYINLSLHYLEQVIIALQEKSMGMNRPHIPYRNSMMTMALKDSLGGNCRTVMVATINSAQDQ
LDESISTCRFAQRVAMVRNTVLLNEELDPSLIIRRLKQELRDLKEEVKMLRGEEEERGPLTPDELVRLQGQVETYVADNSPEASLNLGGSMMMIRAVFEVFKRLLRTGGF
KMAGSGGAGGSGSGAGGGGGEPGTPGRAGGAGEGGGGGGDSAGLQDQVRKLKLQVAQRDNEIGILVSMLKRREGAGAKAGPVLSSPSTSINGPPVPGMGAAAAAGG
AGGGLGAGPSGVDGSGAGPGPGSAAAAGGGGGPADELAVLMNTNLLADRNKAFELFRKSYRQNEVIEENKQLLKTKYDSAKSLGAAVNDSKGRINELRSAIEQRRMQ
RGAAAVAAGMSPEQLTDDDPEESRCKELMEQEKARYRDAFNQLRELKKEIEHLHLLLEQSRTRLQRDFEQWMGLMLRQQQQQQQAAAGLPPSGPSPAMSPMRPSAA
AQQPGMPRQAWGDAGAAPSPSASPAPGPGPSSSGGSLTRGPSPGGSAAAPPPLPGAGGQWATPVGLRASGGNSPALGSHGYGNGHPSGMHGLGPGPGPGPGSGAASPA
VTRTGSGVAVQQHAAAHSSGGPSMQGVDAAVLEMARPFLTGNPDADADIVKFYEAKAKLMQKLSAG

B

Make Database
/ncbi-blast-2.2.29+/bin/makeblastdb -in /Cr_Pp_Kinesin2_Kinesin9.fasta -dbtype prot –out /Cr_Pp_Kinesin2_9_data

Blastx
/ncbi-blast-2.2.29+/bin/blastx -query /REF3Trinity.fasta -db /Cr_Pp_Kinesin2_9_data -num_threads 2 -evalue 1e-100 -outfmt 6 >/ALL_KINESIN2_9.outfmt6

Query Accession	Subject	Percent ID	Alignment length	Mismatches	Gap Openings	Q. Start	Q. End	S. Start	S. End	e-value	Bit score	ID
KT986258	XP_001701617	34.49	603	347	8	335	2011	10	608	5.00E-101	332	CrKLP1
KT986258	Phypa_458410	41.21	381	193	6	323	1453	12	365	8.00E-109	285	PpKinesin-9A
KT986235	Phypa_425592	60.71	700	244	10	289	2313	41	734	0.00E+00	749	PpKinesin-2
KT986235	XP_001701510	45.07	670	317	11	289	2211	17	664	1.00E-156	494	FLA10
KT986235	XP_001697037	60.61	363	131	5	289	1368	14	367	6.00E-143	455	FLA8
KT986259	Phypa_428375	45.65	758	282	11	107	2182	13	706	0.00E+00	597	PpKinesin-9B
