# Supplementary material for: Kinesin-2 and kinesin-9 have atypical functions during ciliogenesis in the male gametophyte of Marsilea vestita
Source: BMC Cell Biol. 2016 Jul 16;17:29. doi: 10.1186/s12860-016-0107-7 (PMC4947347; doi:10.1186/s12860-016-0107-7)
Supplement: Additional file 2: — Building a kinesin-2 and a kinesin-9 phylogenetic tree. Source of kinesin-2 and kinesin-9 motor domains used for multiple sequence alignment (MSA) and to build phylogenetic trees. (PDF 1418 kb) [file 12860_2016_107_MOESM2_ESM.pdf]

A

Analysis ID	Subfamily	Accession Number	Reference	Species
Mv_Kinesin-2	2	KT986235	http://www.ncbi.nlm.nih.gov/genbank/	Marsilea vestita
Am_Kinesin-2A	2A	XP_396164	http://www.ncbi.nlm.nih.gov/genbank/	Apis mellifera
Am_Kinesin-2B	2B	XP_393174	http://www.ncbi.nlm.nih.gov/genbank/	Apis mellifera
Am_Kinesin-2C	2C	XP_395281	http://www.ncbi.nlm.nih.gov/genbank/	Apis mellifera
Ce_KLP-11	2B	NP_001023139	http://www.ncbi.nlm.nih.gov/genbank/	Caenorhabdatis elegans
Ce_KLP-20	2A	NP_497178	http://www.ncbi.nlm.nih.gov/genbank/	Caenorhabdatis elegans
Ce_OSM-3	2C	NP_001023308	http://www.ncbi.nlm.nih.gov/genbank/	Caenorhabdatis elegans
Cr_FLA10	2	XP_001701510	http://www.ncbi.nlm.nih.gov/genbank/	Chlamydomonas reinhardtii
Cr_FLA8	2	XP_001697037	http://www.ncbi.nlm.nih.gov/genbank/	Chlamydomonas reinhardtii
Dm_KIF3C	2C	NP_651939.4	http://www.ncbi.nlm.nih.gov/genbank/	Drosophila melanogaster
Dr_KLP64D	2A	NP_523934.1	http://www.ncbi.nlm.nih.gov/genbank/	Drosophila melanogaster
Dr_KLP68D	2B	NP_524029.2	http://www.ncbi.nlm.nih.gov/genbank/	Drosophila melanogaster
Gg_KIF3B	2B	NP_001012852.1	http://www.ncbi.nlm.nih.gov/genbank/	Gallus gallus
Gl_Kinesin-2D	2D	XP_001708236	http://www.ncbi.nlm.nih.gov/genbank/	Giardia lamblia
Gl_Kinesin-2D	2D	XP_001706504	http://www.ncbi.nlm.nih.gov/genbank/	Giardia lamblia
Hs_KIF17	2C	NP_001116291	http://www.ncbi.nlm.nih.gov/genbank/	Homo sapiens
Hs_KIF3A	2A	NP_008985.3	http://www.ncbi.nlm.nih.gov/genbank/	Homo sapiens
Hs_KIF3B	2B	NP_004789.1	http://www.ncbi.nlm.nih.gov/genbank/	Homo sapiens
Hs_KIF3C	2B	NP_002245	http://www.ncbi.nlm.nih.gov/genbank/	Homo sapiens
Lm_Kinesin-2D	2D	XP_001682337	http://www.ncbi.nlm.nih.gov/genbank/	Leishmania major
Lm_Kinesin-2D	2D	XP_001685383	http://www.ncbi.nlm.nih.gov/genbank/	Leishmania major
Pt_Kinesin-2	2	XP_001455773	http://www.ncbi.nlm.nih.gov/genbank/	Phaeodactylum tricornutum
Pt_Kinesin-2	2	XP_001426973	http://www.ncbi.nlm.nih.gov/genbank/	Phaeodactylum tricornutum
Pt_Kinesin-2	2	XP_001427404	http://www.ncbi.nlm.nih.gov/genbank/	Phaeodactylum tricornutum
Pt_Kinesin-2	2	XP_00142818	http://www.ncbi.nlm.nih.gov/genbank/	Phaeodactylum tricornutum
Pt_Kinesin-2	2	XP_001428184	http://www.ncbi.nlm.nih.gov/genbank/	Phaeodactylum tricornutum
Pt_Kinesin-2	2	XP_001429325	http://www.ncbi.nlm.nih.gov/genbank/	Phaeodactylum tricornutum
Pt_Kinesin-2	2	XP_001429366	http://www.ncbi.nlm.nih.gov/genbank/	Phaeodactylum tricornutum
Pp_Kinesin2	2	Phypa_425592	V1.6_proteins - cosmoss.org	Physcomitrella patens
Sp_KRP85	2A	NP_999777.1	http://www.ncbi.nlm.nih.gov/genbank/	Strongylocentrotus purpuratus
Sp_KRP95	2B	NP_999817.1	http://www.ncbi.nlm.nih.gov/genbank/	Strongylocentrotus purpuratus
Tb_Kinesin-2D	2D	Tb11.01.5490	http://www.genome.jp/	Trypanosoma brucei
Tb_Kinesin-2D	2D	Tb927.5.2090	http://www.genome.jp/	Trypanosoma brucei
Tt_Kinesin-2	2	XP_001014287	http://www.ncbi.nlm.nih.gov/genbank/	Tetrahymena thermophila
Tv_Kinesin-2	2	XP_001276971	http://www.ncbi.nlm.nih.gov/genbank/	Trichomonas vaginalis
Tv_Kinesin-2	2	XP_001300992	http://www.ncbi.nlm.nih.gov/genbank/	Trichomonas vaginalis
Tv_Kinesin-2	2	XP_001315568	http://www.ncbi.nlm.nih.gov/genbank/	Trichomonas vaginalis
Tv_Kinesin-2	2	XP_001319907	http://www.ncbi.nlm.nih.gov/genbank/	Trichomonas vaginalis
Tv_Kinesin-2	2	XP_001579747	http://www.ncbi.nlm.nih.gov/genbank/	Trichomonas vaginalis

B

Analysis ID	Subfamily	Source	Reference	Species
Mv_Kinesin-9A	9A	KT986258	http://www.ncbi.nlm.nih.gov/genbank/	Marsilea vestita
Mv_Kinesin-9B	9B	KT986259	http://www.ncbi.nlm.nih.gov/genbank/	Marsilea vestita
Am_Kinesin-9B	9B	XP_006561916	http://www.ncbi.nlm.nih.gov/genbank/	Apis mellifera
Cr_KLP1	9A	XP_001701617	http://www.ncbi.nlm.nih.gov/genbank/	Chlamydomonas reinhardtii
Cr_Kinesin-9B	9B	Cre01.g036800.t1.1	http://phytozome.jgi.doe.gov/	Chlamydomonas reinhardtii
Gl_Kinesin-9A	9A	XP_001705615	http://www.ncbi.nlm.nih.gov/genbank/	Giardia lamblia
Gl_Kinesin-9B	9B	XP_001707755	http://www.ncbi.nlm.nih.gov/genbank/	Giardia lamblia
Hs_KIF9	9A	NP_071737	http://www.ncbi.nlm.nih.gov/genbank/	Homo sapiens
Hs_KIF6	9B	NP_001275949	http://www.ncbi.nlm.nih.gov/genbank/	Homo sapiens
Lm_Kinesin-9B	9B	XP_001687540	http://www.ncbi.nlm.nih.gov/genbank/	Leishmania major
Pt_Kinesin-9A	9A	XP_001455832	http://www.ncbi.nlm.nih.gov/genbank/	Phaeodactylum tricornutum
Pt_Kinesin-9B	9B	XP_001445877	http://www.ncbi.nlm.nih.gov/genbank/	Phaeodactylum tricornutum
Pt_Kinesin-9B	9B	XP_001430724	http://www.ncbi.nlm.nih.gov/genbank/	Phaeodactylum tricornutum
Pp_Kinesin-9A	9A	Phypa_425498	V1.6_proteins - cosmoss.org	Physcomitrella patens
Pp_Kinesin-9A	9A	Phypa_458410	V1.6_proteins - cosmoss.org	Physcomitrella patens
Pp_Kinesin-9B	9B	Phypa_428375	V1.6_proteins - cosmoss.org	Physcomitrella patens
Tb_Kinesin-9A	9A	Tb927.7.6290	http://www.genome.jp/	Trypanosoma brucei
Tb_Kinesin-9B	9B	Tb927.7.726	http://www.genome.jp/	Trypanosoma brucei
Tt_Kinesin-9B	9B	XP_001025897	http://www.ncbi.nlm.nih.gov/genbank/	Tetrahymena thermophila
Tt_Kinesin-9B	9B	XP_00102480	http://www.ncbi.nlm.nih.gov/genbank/	Tetrahymena thermophila
Tv_Kinesin-9B	9B	XP_001325581	http://www.ncbi.nlm.nih.gov/genbank/	Trichomonas vaginalis
