# Supplementary material for: Kinesin-2 and kinesin-9 have atypical functions during ciliogenesis in the male gametophyte of Marsilea vestita
Source: BMC Cell Biol. 2016 Jul 16;17:29. doi: 10.1186/s12860-016-0107-7 (PMC4947347; doi:10.1186/s12860-016-0107-7)
Supplement: Additional file 11: — Primers and dsRNA constructs. Primers for MvKinesin-2, MvKinesin-9A, MvKinesin-9B, and MvCentrin were generated from the gametophyte transcriptome for (A) RT-PCR and (B) to make dsRNA. (C) Full dsRNA constructs for RNAi. (PDF 564 kb) [file 12860_2016_107_MOESM11_ESM.pdf]

A

MvKinesin-2	Sequence (5'->3')
Forward primer	AAATCAAGAATCCGAGCCAA
Reverse primer	ATTTCAGTCCTTAGAGCAGC
**MvKinesin-9A**	**Sequence (5'->3')**
Forward primer	GTAGGAGTGGGAGATGAGAA
Reverse primer	AAGTTGCTCTTGTTCGTGTA
**MvKinesin-9B**	**Sequence (5'->3')**
Forward primer	TGTCCAGCAAATTAGAACGA
Reverse primer	CTAGGAGTAGGAGCCGATAA
**MvCentrin**	**Sequence (5'->3')**
Forward primer	AGGCCTGAGTGAGGAACAGAAACA
Reverse primer	TCCTTTGCATCAATGGTGCCTGAC

B

MvKinesin-2	Sequence (5'->3')
Forward primer	TAATACGACTCACTATAGGGAAATCAAGAATCCGAGCCAA
Reverse primer	TAATACGACTCACTATAGGGATTTCAGTCCTTAGAGCAGC
**MvKinesin-9A**	**Sequence (5'->3')**
Forward primer	TAATACGACTCACTATAGGGGTAGGAGTGGGAGATGAGAA
Reverse primer	TAATACGACTCACTATAGGGAAGTTGCTCTTGTTCGTGTA
**MvKinesin-9B**	**Sequence (5'->3')**
Forward primer	TAATACGACTCACTATAGGGTGTCCAGCAAATTAGAACGA
Reverse primer	TAATACGACTCACTATAGGGCTAGGAGTAGGAGCCGATAA

C

>MvKinesin-2 dsRNA

TAATACGACTCACTATAGGGAAATCAAGAATCCGAGCCAATGCATTCTGAAAGCCGTATACAAGAGAACAAAACCATTGACGAGGAACGTCAAC
GCAAAATAGCTGAACTAGAGGAAGCACAACTTATGGCTGAAGAAAAATGCAGTACAATGGAGGAAGAGTTAGAATCAAAGACCCGCAAGTTGAG
AAGGCTGATGTCACGCTATCAGCAAAGCAAACTTGATGTTGCTGCTCTAAGGACTGAAATCCCTATAGTGAGTCGTATTA

>MvKinesin-9A dsRNA

TAATACGACTCACTATAGGGGTAGGAGTGGGAGATGAGAAGAAAAGAAATGCGGCATTCCTTGATTTCAAGCATAACACATCTGAAGGCACCCA
GATCTGTTATGTACTGAAAGCAAAAAAGGCAGAACTGAAAGAGAAAAGACTTGCCGTACAGACCCTGCACGATCGAATGAAAGACTGCAAACGG
CAAATTCATGACATAAATTGCCTTCTGATGACAGATAGTCAAATTGATAGTGCACAAGATCAGTCAGATGTAGAGCTGAGCAACAAATTACA
CGAACAAGAGCAACTTCCCTATAGTGAGTCGTATTA

>MvKinesin-9B dsRNA

TAATACGACTCACTATAGGGTGTCCAGCAAATTAGAACGATTTTTTCAATATTCAAGGATATGATAAACAATGGGGGCAATAAAAACACTAAAGA
AGAACATGAAGAAGGAGAAATGAATCCAGAGGAGGATACTAATATGCTTAATGAAACCATACGTGACCTACACCGAAAGATTCAAGAGAGAGAT
AATGAAATCAACATACTAGTTAACTTTATACGAAAGCAAAACCTGGGATGCTTGAAGTCCTTTGAAGGACTAAGAGAAGAATTATCGGCTCCTAC
